# Reversible atrial fibrillation following Crotalinae envenomation

**DOI:** 10.1186/s40409-017-0108-9

**Published:** 2017-03-21

**Authors:** Dan Quan, Kenneth Zurcher

**Affiliations:** 1 Department of Emergency Medicine, Maricopa Integrated Health System, 2601 East Roosevelt Road, Phoenix, AZ 85008 USA; 2 Department of Emergency Medicine, University of Arizona College of Medicine – Phoenix, 550 East Van Buren Street, Phoenix, AZ 85004 USA; 3 University of Arizona College of Medicine – Phoenix, 550 East Van Buren Street, Phoenix, AZ 85004 USA

**Keywords:** Crotalidae, Rattlesnake, Bite, Envenomation, Snakebite, Atrial fibrillation

## Abstract

**Background:**

Cardiotoxicity is a documented complication of Crotalinae envenomation. Reported cardiac complications following snake envenomation have included acute myocardial infarction, electrocardiogram abnormalities and arrhythmias. Few reports exist describing arrhythmia induced by viper envenomation and to our knowledge none describe arrhythmia induced by Crotalinae envenomation. This report concerns the first known case of atrial fibrillation precipitated by rattlesnake bite.

**Case presentation:**

A 73-year-old Caucasian man with a past medical history of hypertension, hyperlipidemia, type 1 diabetes mellitus, and a baseline first-degree atrioventricular block presented to the emergency department following a rattlesnake bite to his left lower leg. He developed pain and swelling in his left leg two-hour post-envenomation and subsequently received four vials of Crotalidae polyvalent immune fab (ovine). At three-hour post-envenomation following transfer to the intensive care unit, an electrocardiogram revealed new-onset atrial fibrillation. An amiodarone drip was started and the patient successfully converted to normal sinus rhythm approximately six hours after he was found to be in atrial fibrillation. A transthoracic echocardiogram revealed mild concentric left ventricular hypertrophy and an ejection fraction of 72%. He was discharged the following day with no hematological abnormalities and a baseline first-degree atrioventricular block.

**Conclusion:**

This is the first documented case of reversible atrial fibrillation precipitated by Crotalinae envenomation. In patients with pertinent risk factors for developing atrial fibrillation, physicians should be aware of the potential for this arrhythmia. Direct toxic effects of venom or structural and electrophysiological cardiovascular abnormalities may predispose snakebite patients to arrhythmia, warranting extended and attentive cardiac monitoring.

## Background

Endemic to Asia and the Americas, the Viperidae subfamily of snakes known as Crotalinae is comprised of rattlesnakes, cottonmouths, and copperheads in North America; *Bothrops* in Central and South America; and the Asiatic pit vipers [1, 2]. Clinical manifestations of Crotalinae envenomation often include local tissue reaction with systemic effects such as nausea, vomiting, diarrhea, diaphoresis, and weakness. Severe cases are characterized by coagulopathy with hemorrhagic manifestations, as well as renal failure [3]. While the potential nephrotoxic, myotoxic, and neurotoxic effects of Viperidae family snake venom are well documented, cardiotoxicity is infrequently described in the literature. Reported cardiac complications following snake envenomation have included acute myocardial infarction, electrocardiogram (ECG) abnormalities, and arrhythmias [4–16]. Few reports exist describing arrhythmia induced by viper envenomation, and to our knowledge none describe arrhythmia induced by Crotalinae envenomation. This report concerns a case of atrial fibrillation (AF) precipitated by rattlesnake bite.

## Case presentation

A 73-year-old Caucasian man presented to the emergency department following a rattlesnake bite to his left lower leg. The envenomation occurred 30 min prior to his arrival by ambulance while he was retrieving a golf ball at a local golf course. On presentation, the patient was alert, complained of pain localized to the left lower extremity, and denied having chest pain, palpitations or dyspnea. His past medical history included hypertension, hyperlipidemia and type 1 diabetes mellitus. Current medications included lisinopril, hydrochlorothiazide, atorvastatin, fluoxetine and insulin. On examination, two puncture wounds and mild swelling were noted anteriorly on the patient’s left lower leg.

An ECG taken approximately one hour after envenomation showed him to be in sinus rhythm with a rate of 87 beats per minute. Additionally, a PR interval of 220 ms was indicative of a baseline first-degree atrioventricular block (AVB). Initial laboratory tests indicated a number of abnormal values: WBC 11,000/μL (H), neutrophils 75.2% (H), lymphocytes 19.7% (L), D-dimer 0.55 μg/mL (H), glucose 222 mg/dL (H), BUN 34 mg/dL (H). The following laboratory values were in the normal range: platelets 312 × 10^3^/μL, prothrombin time 12.9 s and fibrinogen 305 mg/dL.

Two hours post-envenomation the patient developed moderate swelling in his left calf and leg, tachycardia, nausea and diaphoresis. At that time, he was started on an initial dose of four vials of Crotalidae polyvalent immune fab (ovine) infused over one hour.

**Fig. 1:**
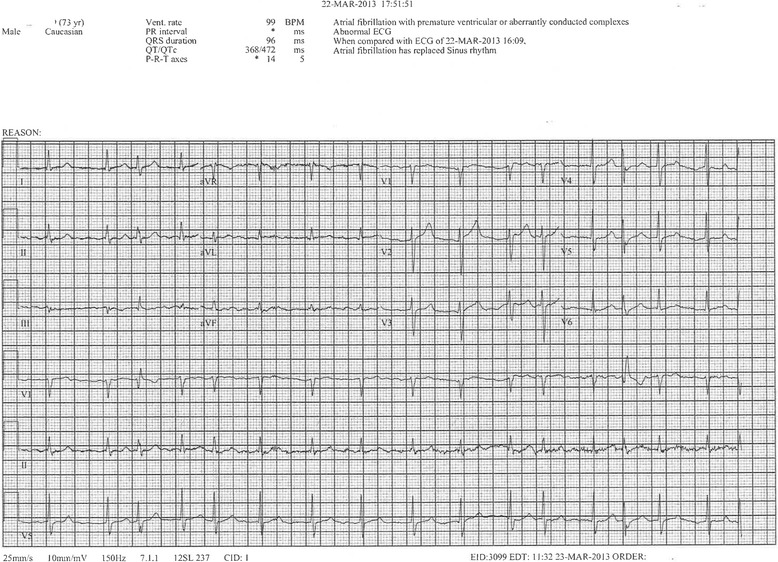
Electrocardiogram obtained three hours post pit viper envenomation

**Fig. 2:**
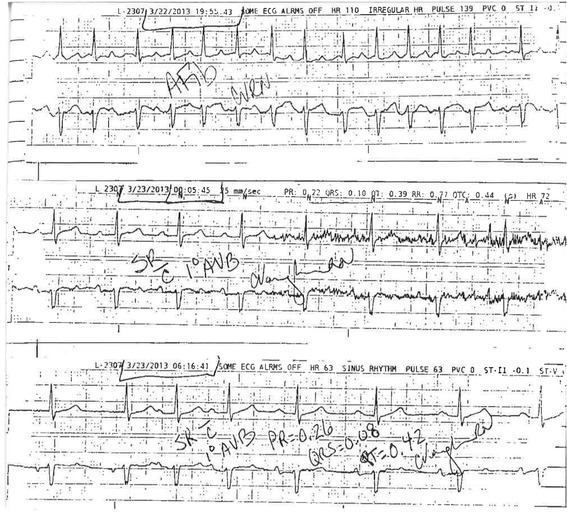
Rhythm strips showing conversion from atrial fibrillation to normal sinus rhythm

The patient was subsequently transferred to the ICU for monitoring, however an ECG taken three hours post-envenomation revealed new-onset AF (Fig. 1). Cardiology team was consulted and an amiodarone drip was started. The patient successfully converted to normal sinus rhythm approximately six hours after he was found to be in AF (Fig. 2). A first-degree AVB was still present at that time. A transthoracic echocardiogram was ordered by cardiology and revealed mild concentric left ventricular hypertrophy with normal systolic function and an ejection fraction of 72%. The patient denied use of any stimulants or sympathomimetic agents, as well as any family history of heart disease. TSH levels were within normal limits.

The patient’s blood sugar was markedly elevated during the first 24 h of hospitalization, and his insulin regimen was adjusted accordingly. No further antivenom was given as his swelling and laboratory tests were well controlled. Coagulation studies were monitored, and the patient continued to be stable for the rest of his hospitalization. His laboratory studies prior to discharge were stable. He was discharged the following day with ecchymosis around the ankle and wound site and instructions to have his blood work repeated at an outside laboratory.

## Discussion

In 2014, over 3,000 Crotalinae envenomations were reported to the American Association of Poison Control Centers’ National Poison Data System. Of those envenomations, over 750 were attributed to rattlesnakes [17]. According to one Indian study assessing cardiac complications of snakebite, it is estimated that up to 25% of viper family envenomations result in cardiotoxicity [10].

Cardiovascular manifestations of snakebite, particularly of the Viperidae family variety, are diverse. Reports of myocardial infarction and cardiac tamponade have been described in the literature, and ECG abnormalities such as T-wave inversion and QT prolongation have been seen in several cases [4–10, 18]. Arrhythmia is a rare complication of snakebite and has been described infrequently; reported arrhythmias following viper envenomation include ventricular tachycardia, torsades de pointes, second-degree AVB, and sinus arrest [9, 11–13]. In reports nonspecific to Crotalidae or Viperidae envenomation, cases of first-degree AVB, bundle branch block, atrial arrhythmias, complete heart block, sinus bradycardia or tachycardia, and various sinus arrhythmias have also been described [10, 14, 15]. To our knowledge only one case of confirmed reversible AF following snakebite exists in the literature, however the species of snake was unknown [16]. The exact mechanism responsible for snakebite-induced arrhythmia remains unclear.

Several theoretical factors may have contributed to the development of paroxysmal AF in our patient. Given his cardiovascular risk factors, a predisposition towards arrhythmia likely existed prior to envenomation. Advanced age, hyperlipidemia, long-standing type 1 diabetes mellitus and hypertension are established extracardiac predictors of AF [19]. Mild left ventricular hypertrophy, seen on this patient’s transthoracic echocardiogram and likely secondary to his underlying hypertension, is also associated with an increased risk [20].

Potential atrial electrical abnormalities may represent another mechanism behind this patient’s development of AF. In a 2014 retrospective study by Uhm et al. [21], first-degree AVB was found to be an independent risk factor (hazard ratio 2.33) for development of AF. Autonomic nervous system activation and subsequent adrenergic stimulation following envenomation also could have triggered AF. Through several proposed electrophysiological mechanisms in the atrium, catecholamines have been shown to affect AF initiation and/or maintenance [22].

Additionally, arrhythmia precipitated by direct toxicity to Crotalidae venom must be considered. Snake venom is believed to have a potential toxic effect on the myocardium, and may modify electrophysiological properties of the cardiac cell membrane [4, 5, 11]. This could potentially alter cardiac impulse generation and/or conduction; however, a mechanism remains to be determined.

## Conclusion

This report describes the case of a 73-year-old man who developed reversible atrial fibrillation following Crotalinae envenomation. Although this is a rare presentation of snakebite, in patients with pertinent risk factors for developing AF, physicians should be aware of the potential for this arrhythmia. Direct toxic effects of venom or structural and electrophysiological abnormalities may predispose snakebite patients to arrhythmia, warranting extended and attentive cardiac monitoring.

## Abbreviations

AF: Atrial fibrillation
AVB: Atrioventricular block
BUN: Blood urea nitrogen
ECG: Electrocardiogram
ICU: Intensive care unit
TSH: Thyroid-stimulating hormone
WBC: White blood cell count
